# The performance of large language models in intercollegiate Membership of the Royal College of Surgeons examination

**DOI:** 10.1308/rcsann.2024.0023

**Published:** 2024-03-06

**Authors:** J Chan, T Dong, GD Angelini

**Affiliations:** Bristol Heart Institute, University of Bristol, UK

**Keywords:** Large language models, Bard, MRCS, ChatGPT

## Abstract

**Introduction:**

Large language models (LLM), such as Chat Generative Pre-trained Transformer (ChatGPT) and Bard utilise deep learning algorithms that have been trained on a massive data set of text and code to generate human-like responses. Several studies have demonstrated satisfactory performance on postgraduate examinations, including the United States Medical Licensing Examination. We aimed to evaluate artificial intelligence performance in Part A of the intercollegiate Membership of the Royal College of Surgeons (MRCS) examination.

**Methods:**

The MRCS mock examination from Pastest, a commonly used question bank for examinees, was used to assess the performance of three LLMs: GPT-3.5, GPT 4.0 and Bard. Three hundred mock questions were input into the three LLMs, and the responses provided by the LLMs were recorded and analysed. The pass mark was set at 70%.

**Results:**

The overall accuracies for GPT-3.5, GPT 4.0 and Bard were 67.33%, 71.67% and 65.67%, respectively (*p* = 0.27). The performances of GPT-3.5, GPT 4.0 and Bard in Applied Basic Sciences were 68.89%, 72.78% and 63.33% (*p* = 0.15), respectively. Furthermore, the three LLMs obtained correct answers in 65.00%, 70.00% and 69.17% of the Principles of Surgery in General questions (*p* = 0.67). There were no differences in performance in the overall and subcategories among the three LLMs.

**Conclusions:**

Our findings demonstrated satisfactory performance for all three LLMs in the MRCS Part A examination, with GPT 4.0 the only LLM that achieved the pass mark set.

## INTRODUCTION

ChatGPT, also known as Chat Generative Pre-trained Transformer, is a large language model (LLM) for natural language processing developed by OpenAI (San Francisco, CA, US). It utilises deep learning algorithms trained on a massive data set of text and code to generate human-like responses. ChatGPT (GPT-3.5) has received considerable attention since its release in November 2022. GPT 4, an upgraded version of ChatGPT, was released in March 2023 with the aim of handling more nuanced instructions than GPT-3.5. Bard, another LLM, was released by Google (Mountain View, CA, US) five months later.

Several studies have examined the performance of ChatGPT in postgraduate medical examinations. ChatGPT was close to or above the pass threshold in the United States Medical Licensing Examination (USMLE), Canadian Royal College and American Board of Radiology examinations, American Board of Thoracic Surgery, and Neurosurgery Written Board Examinations.^1–5^

We aimed to evaluate the performance of LLM in the intercollegiate Membership of the Royal College of Surgeons (MRCS).

## Methods

Pastest, a commonly used question bank for junior doctors preparing for the MRCS examination, was used to examine the performance of the three LLMs. The level of difficulty of all individual questions (easy, average and difficult), peer performance and results based on subspecialities were available in the feedback session after completion of the mock exam. The pass mark for the mock examination was preset to 210/300 (70%) by Pastest.

**Figure 1:**
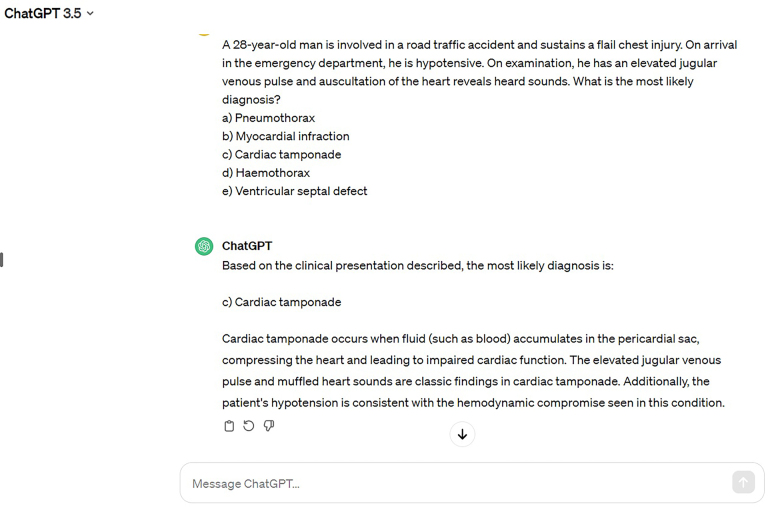
Question input into the large language model (GPT-3.5 in this example) and its responses

The three LLMs used in this study were GPT-3.5 (OpenAI) May 12 version, GPT 4 (OpenAI) and the Bard Experiment (Google). All 300 questions were input into the LLM, which was performed in May 2023. The questions were formatted with the question text followed by the direct question and a prompt line of “Please choose the best option from below”. Questions with images were not included in this study because GPT-3.5 accepts only textual input. The responses provided by the LLM were recorded and analysed. Figure 1 shows an example of a question prompt and response by GPT-3.5.

### Ethical statement

No data related to patient information were used in this study. The question bank and part of the information underlying this article were provided by Pastest, UK, with permission. Data will be shared on request with the corresponding author with permission from Pastest UK. The study was performed in accordance with the ethical standards of the 1964 Declaration of Helsinki and its later amendments. General Data Protection Regulations were strictly followed for all data.

### Statistical analysis

Continuous variables were reported as mean and standard deviation. Categorical variables were reported as frequencies and percentages.

The Kruskal–Wallis test was used to compare categorical categories among the three groups after the Shapiro–Wilk normality test demonstrated that the data were not normally distributed. The post hoc Dunn’s test was used to conduct pairwise comparisons among the independent groups if the Kruskal–Wallis test was statistically significant. Statistical significance was defined as *p* < 0.05.

R (version 4.1.1) and R Studio (version 1.4.1103, RStudio, PBC) were used to perform statistical analyses. The tidyverse and MultNonParam packages were used. Graphs and tables were created using Microsoft Office 365 (version 16.0.14026, 64 bit).

## Results

### Overall performance

**Figure 2:**
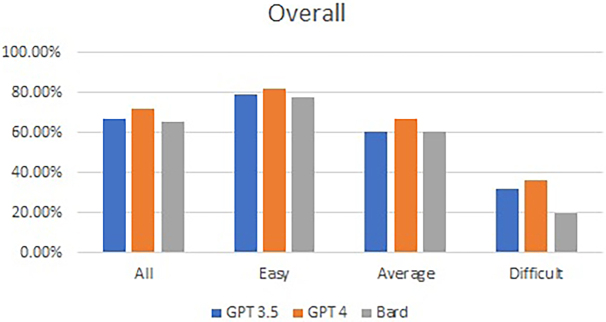
Overall performance and subcategorical performance based on the level of difficulty of all 300 mock questions for the three large language models

The overall accuracies for GPT-3.5, GPT 4.0 and Bard were 67.33%, 71.67% and 65.67%, respectively. There was no statistical difference in performance among the three LLMs (*p* = 0.27). Practically, GPT 4.0 passed the test, whereas the other two LLMs failed. The performance of the three LLMs decreased as the level of difficulty increased, from a maximum score of 82.19% on GPT 4 for easy questions to 20% for difficult questions. Figure 2 shows the overall and subcategorical performances of the three LLMs based on the difficulty of the questions.

### Applied Basic Sciences and Principles of Surgery in General

**Figure 3:**
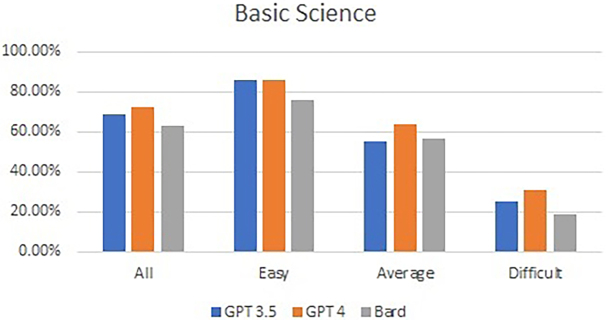
Overall performance and subcategorical performance of all three large language models in Applied Basic Sciences

**Figure 4:**
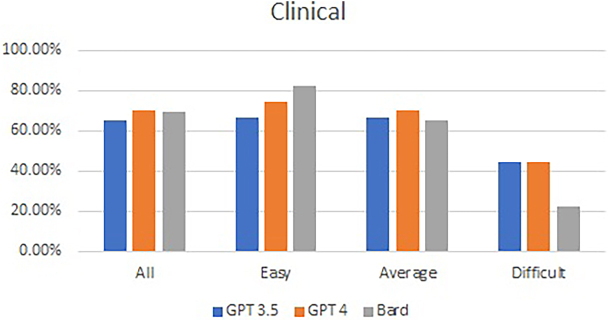
Overall and subcategorical performance of all three large language models in Principles of Surgery in General

No statistical differences were observed in the performance of GPT-3.5, GPT 4.0 and Bard in the Applied Basic Sciences mock examination (68.89%, 72.78%, and 63.33%, respectively; *p* = 0.15) or in the Principles of Surgery in General (65.00%, 70.00%, and 69.17%, respectively; *p* = 0.67). Practically, GPT 4.0 passed the test, whereas the other two LLMs failed. A similar trend was noted, with a reduction in performance as the level of difficulty increased. Figures 3 and 4 show the overall and subcategorical performances of all three LLMs based on the difficulty of the questions in the Applied Basic Sciences and Principles of Surgery in General mock paper.

### Performance in individual category

As an upgraded version of GPT-3.5, the accuracy of GPT 4 is better in all subcategories in both the Applied Basic Sciences and Principles of Surgery in General mock papers. The total performance of Bard is similar to that of GPT 4 in the Principles of Surgery in General (83/120 and 84/120), although it is noticeably lower in Surgical Anatomy compared with both GPT-3.5 and GPT 4 (55/80, 57/80, and 45/80). Tables 1 and 2 summarise the performance of the LLM in subsections in Applied Basic Sciences and the Principles of Surgery in General mock examination.

**Table 1:** Performance of the large language model in subsections of the Applied Basic Sciences mock examination

Applied Basic Sciences	GPT-3.5	GPT 4	Bard	Total no. of questions
Applied Surgical Anatomy raw score	55	57	45	80
Physiology + Pharmacology raw score	32	34	32	45
Pathology + Microbiology raw score	37	40	37	55
Total correct answer	124	131	114	180

**Table 2:** Performance of the large language model in subsections of the Principles of Surgery in General mock examination

Principles of Surgery in General	GPT-3.5	GPT 4	Bard	Total no. of questions
Common Surgical Conditions	34	36	36	50
Perioperative Management	27	29	28	40
Trauma	17	19	19	30
Total correct answer	78	84	83	120

## Discussion

Our findings demonstrated satisfactory performance for all three LLMs in the MRCS Part A examination. GPT 4.0 was the only LLM that achieved the pass mark set for the mock examination; the other two LLMs were very close (<5% difference) to the pass mark (70%).

### MRCS examination

The MRCS Part A is a 5 hour examination with 300 single best answer questions and is divided into two parts: Applied Basic Sciences (180 questions, 3 hours) and Principles of Surgery in General (120 questions, 2 hours). Applied Basic Sciences focuses on surgical anatomy, physiology and pathology, whereas Principles of Surgery in General focuses on the perioperative area of all surgical specialties. MRCS Part A can be taken in multiple locations in the United Kingdom via computer-based testing centres, with a maximum of six attempts allowed. Doctors who wish to pursue high surgical training must pass the MRCS (both parts A and B) before starting.

### Large language model

LLMs are a form of artificial intelligence (AI) that undergo training on extensive data sets of text and code, enabling them to generate text, translate languages, create various types of creative content and provide informative answers to queries. Among the most widely used LLMs is GPT-3.5, developed by OpenAI, which boasts 175 billion parameters, the largest of any LLM, enabling it to generate text that is more realistic and coherent than its predecessors. Another popular LLM still under development is GPT 4, which is expected to surpass GPT-3.5 with 100 times more parameters to make it even more powerful.^6^

Google AI has also released a new LLM, Bard, which is trained on vast data sets of text and code, and possesses the ability to generate text, translate languages, create diverse creative content and provide informative answers to queries. LLMs remain at the developmental stage but may have the potential to assist with medical education and, potentially, even in clinical decision-making.

### LLM performance in postgraduate medical examination

Several studies have examined the performance of LLMs, in particular GPT-3.5 and GPT 4, in undergraduate and postgraduate medical examinations. Kung *et al* demonstrated that ChatGPT performed at or near the pass threshold for all three parts (steps 1, 2 and 3) of the USMLE.^5^ Gilson *et al* performed similar studies using some of the commonly used question banks (AMBOSS) for USMLE. ChatGPT (GPT 3.5) has achieved accuracies of 44–60%.^4^ Both studies concluded that ChatGPT can achieve the equivalent of a passing score for medical students and the ability to provide logic and informational context across the majority of answers.

In terms of postgraduate examinations, studies have examined the performance of ChatGPT in Canadian Royal College and American Board of Radiology examinations, American Board of Thoracic Surgery, and Neurosurgery Written Board Examinations.^1,3,7^ GPT-3.5 and GPT 4 achieved scores of 73.4% and 83.4% (passing threshold: 69%) in the 500-question mock US neurosurgical written board examination compared with the average candidate score of 73.7%.^1^ Similar results (69%) were observed in the study by Bhayana *et al*, who designed 150 multiple-choice questions that matched the style, content and difficulty of the Canadian Royal College and American Board of Radiology examinations.^3^ Finally, Antaki *et al* examined the performance of ChatGPT in the American Ophthalmic Knowledge Assessment Program examination, with an accuracy of 42.7–55.8%.^2^

LLMs may even have the potential to break language barriers and improve the performance of postgraduate students. A study by Liu *et al* found that ChatGPT was equally effective in the Clinical Medicine Entrance Examination, in which the exam is written in Chinese. GPT-3.5 students were able to pass the exam and provide multiple insights into the Chinese language.^8^

### Limitation

This study had several limitations. First, although the mock test is specifically designed for the MRCS examination, the question style may not completely reflect the exact questions in the examination. Moreover, the questions are constantly updated to reflect the syllabus and evolution of the surgical field. None of the three LLMs was designed specifically to answer medical questions, and no research articles and information retrieval tools, such as Google Scholar, UpToDate, DynaMed or PubMed, were used in the original code. Despite its limitations, ChatGPT is a promising tool for medical education. The performance would be further enhanced with further ‘training’ by the inclusion of medical research articles and textbooks.

## Conclusions

The performance of all three LLMs in Part A of the MRCS examination was satisfactory. However, GPT 4.0 was the only LLM to achieve the pass mark set for the mock examination. Further work should be conducted to integrate LLMs to improve the learning of postgraduate students while objectively assessing their examination performance.

## Data availability

The data underlying this article were provided by Pastest by permission. Data will be shared on request to the corresponding author with the permission of Pastest.

## Author contributions

JC: conceptualisation, data curation, formal analysis, methodology, writing – original draft, and writing – review and editing. TD: data curation, formal analysis, methodology, and writing – review and editing. GDA: conceptualisation, supervision, methodology, and writing – review and editing.
